# Supplementary material for: DMRforPairs: identifying Differentially Methylated Regions between unique samples using array based methylation profiles
Source: BMC Bioinformatics. 2014 May 15;15:141. doi: 10.1186/1471-2105-15-141 (PMC4046028; doi:10.1186/1471-2105-15-141)
Supplement: Additional file 2 — DMRforPairs output for the comparison of A431-MCF7 and NA17018-NA17105. Please start from the HTML files in each folder. Available via the BMC Bioinformatics website. [file 1471-2105-15-141-S2.zip › 1394847754114233_MOESM2_ESM/A431_MCF7/figures/6402.pdf]

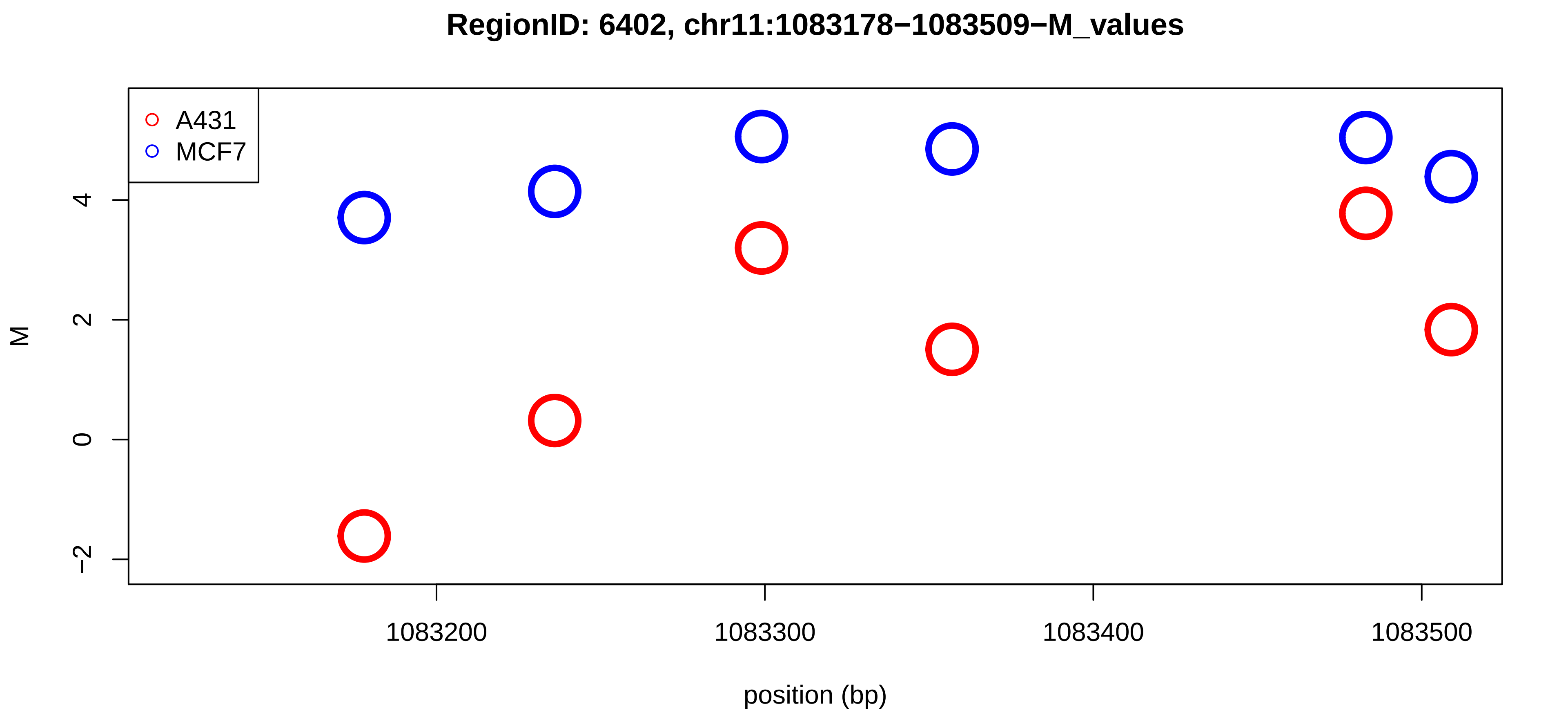
RegionID: 6402, chr11:1083178−1083509−M_values
A431
MCF7
M
−2
0
2
4
1083200
1083300
1083400
1083500
position (bp)

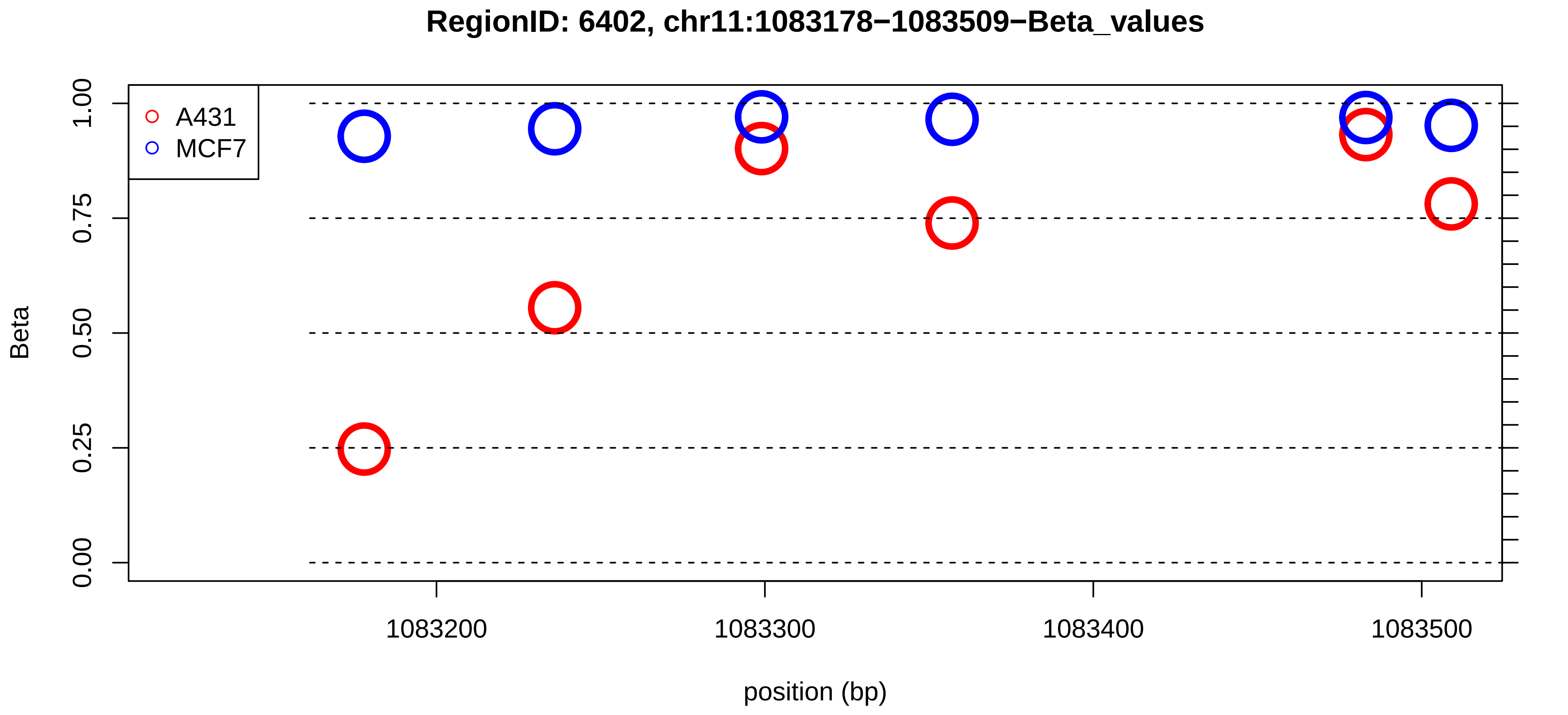
RegionID: 6402, chr11:1083178−1083509−Beta_values
A431
MCF7
Beta
0.00
0.25
0.50
0.75
1.00
1083200
1083300
1083400
1083500
position (bp)

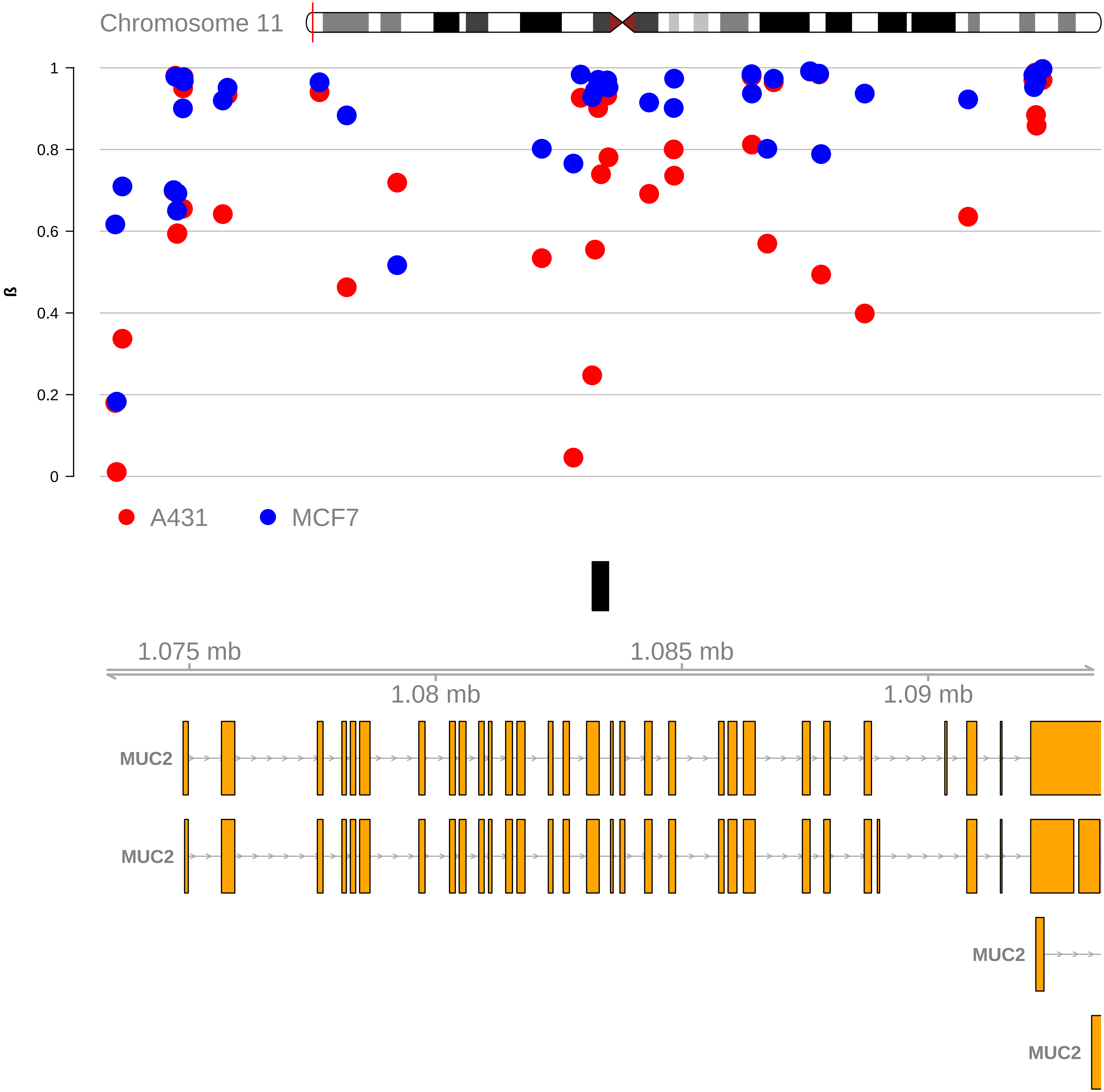

Chromosome 11
β
1
0.8
0.6
0.4
0.2
0
A431
MCF7
1.075 mb
1.085 mb
1.08 mb
1.09 mb
MUC2
MUC2
MUC2
MUC2
